# The mucosal immune system and IgA nephropathy

**DOI:** 10.1007/s00281-021-00871-y

**Published:** 2021-10-12

**Authors:** Loreto Gesualdo, Vincenzo Di Leo, Rosanna Coppo

**Affiliations:** 1 Nephrology, Dialysis and Transplantation Unit, Department of Emergency and Organ Transplantation, University of Bari Aldo Moro, Bari, Italy; 2 Fondazione Ricerca Molinette, Regina Margherita Hospital, Turin, Italy

**Keywords:** IgA nephropathy, Mucosal immunity, Gut-kidney axis, Tonsil-kidney axis, Microbiota, Diet

## Abstract

The precise pathogenesis of immunoglobulin A nephropathy (IgAN) is still not clearly established but emerging evidence confirms a pivotal role for mucosal immunity. This review focuses on the key role of mucosa-associated lymphoid tissue (MALT) in promoting the onset of the disease, underlying the relationship among microbiota, genetic factors, food antigen, infections, and mucosal immune response. Finally, we evaluate potential therapies targeting microbes and mucosa hyperresponsiveness in IgAN patients.

## Introduction

IgAN is the commonest primary glomerulonephritis (GN) worldwide, which determines the end-stage kidney disease (ESKD) in 20–40% of cases [1]. The connection between IgAN and the mucosal-associated immune system has been contemplated shortly after the discovery of this glomerular disease [2] because IgA, which is the predominant class of immunoglobulins in renal deposits, is mostly produced by mucosa-associated lymphoid tissue (MALT) and is prevalent in mucosal secretions. This hypothesis has been further supported by the clinical characteristic of IgAN, the presentation with macroscopic hematuria simultaneous with upper respiratory tract, or other mucosal infections [3, 4].

IgAN is assumed to be due to glomerular deposition of hypogalactosylated IgA1 (Gd-IgA1). The formation of autoantibodies IgG or IgA directed versus Gd-IgA1 is caused by the synthesis of polymeric Gd-IgA1 (first step), and it is followed by the circulation of immune complexes (IgA-CIC). Moreover, the IgA1 can bind to the IgA Fc receptor (CD89/FcαRI), expressed by myeloid cells, inducing the release of soluble CD89 and creating IgA-sCD89 immunocomplexes [5]. These may accumulate in the mesangium, stimulating the production of cytokines and chemokines, the promotion of inflammation, and finally determining a renal injury of IgAN [6].

The direct or indirect implication of mucosal immunity in the development and progression of the disease has been explored over the last decades by a multitude of studies, each providing a tile to a complex network which is however only partially identified. The insights into this area are particularly valuable not only for the understanding of the pathogenetic events operating in IgAN but most of all for the perspective to new targeted therapeutic approaches.

## Immunoglobulin A nephropathy

### Immunoglobulin A and IgA nephropathy

The pathogenetic key factor in IgAN is deregulated glycosylation of IgA molecule, which particularly affects the highly glycosylated IgA1 subclass. IgA1 presents with the unique insertion of six short O-linked oligosaccharide chains, made by a core of N-acetyl galactosamine (GalNAc) with β1,3-linked galactose (Gal). The addition of galactose to these GalNAc residues is catalyzed by the core 1 synthase, glycoprotein-N-acetyl galactosamine 3-beta-galactosyltransferase, 1 (also known as C1GalT1), which requires the specific chaperone Cosmc (core 1 β3GalT specific molecular chaperone). The galactose and/or the GalNAc residues may be sialylated, respectively, with α 2, 3-linked and α 2, 6-linked sialic acid. In IgAN patients, some GalNAc residues could be early sialylated, preventing the addition of the galactose [7].

The Gd-IgA1 molecule can self-aggregate or form IgA-CIC with autoreacting IgG antibodies [8, 9].

**Fig. 1:**
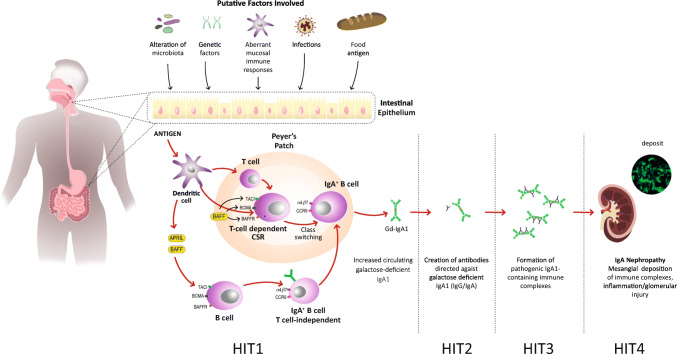
The formation of Gd-IgA1 is the initial hit in the pathogenesis of IgAN; indeed, it can take action as an autoantigen leading to the synthesis of autoantibodies (IgG-IgA: second hit). The creation of immunocomplexes (ICs) and the deposition of these in the kidney have been described to provoke cellular proliferation and inflammation, leading to kidney damage (third and fourth hits) [6, 7]

Notably, IgA in general and Gd-IgA1 in particular have innate-like recognition properties via sugar-mediated lectin binding including binding to alternative and lectin complement pathway components and to fibronectin, laminin, and collagen which may favor the attraction and binding of Gd-IgA1 to the mesangial matrix and local complement activation. IgA1 in glomerular deposits of subjects with IgAN is polymeric, hence as detailed below of mucosal origin. The formation of polymeric hypogalactosylated IgA1 is the first stage in the “multi-hit pathogenesis” of IgAN, and these are produced after mucosal antigenic challenge [5, 10–12] (Fig. 1) and Table 1.

**Table 1:**
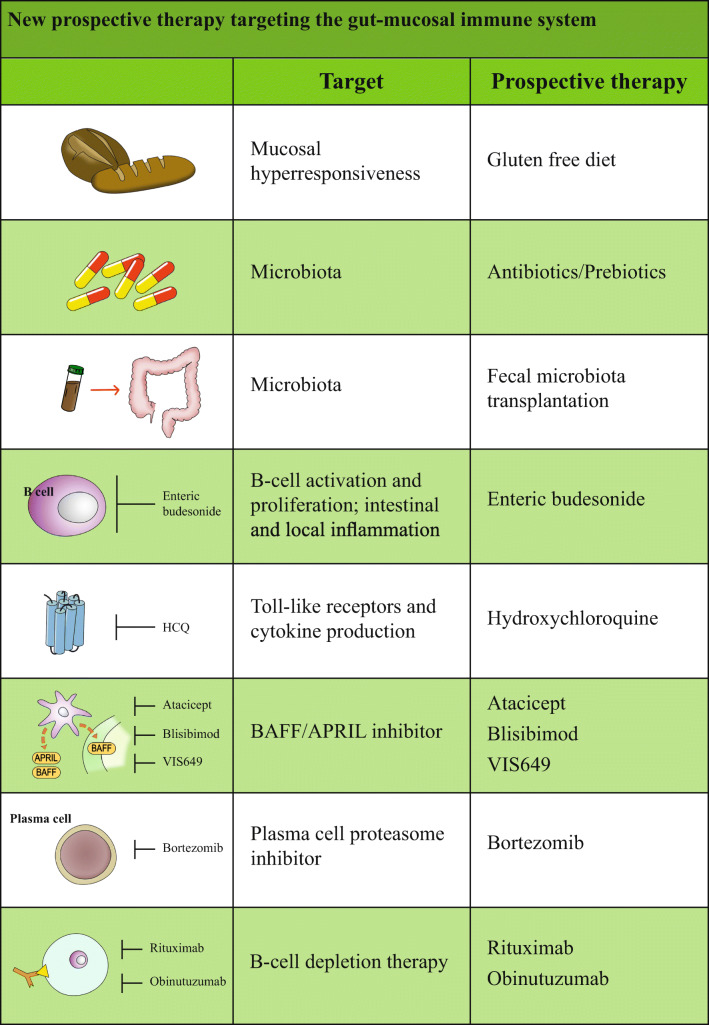
New prospective therapy targeting the gut-mucosal immune system

### The mucosal origin of hypogalactosylated IgA1 in IgAN

IgA is the typical product of MALT. About half of the all lymphocytes are situated at the MALT along the mucosal surfaces which form a selectively permeable barrier in contact with the microbiota [13]. The major function of MALT is the defense against environmental microbes and induction of immunotolerance [14, 15]. MALT is represented in various body areas. The gut-associated lymphoid tissue (GALT) and the nasopharynx-associated lymphoid tissue (NALT) are considered to be implicated in IgAN pathogenesis and progression.

From a quantitative point of view, GALT covers a surface of 230–300 m^2^ throughout the intestine and is one of the largest lymphoid organs. It involves both isolated and grouped lymphoid follicles, Peyer’s patches (PPs), mostly found in the small intestine, mainly in the distal jejunum and the ileum [16]. The follicle-associated epithelium (FAE) surrounds the lymphoid follicles and separates the GALT from the luminal environment. In the FAE are located specialized cells, the so-called M cells, responsible for the uptake of luminal food or environmental antigens. These antigens may reach the underlying immune cells, causing an activation or inhibition of the immune response [17].

The NALT is collectively called Waldeyer’s tonsillar ring, of which palatine and pharyngeal tonsils are the major components. The oral-nasal cavity, as the initial portion of the upper respiratory tract, protects from the intrusion of pathogenic microbes into the mouth and has an important function as an immune organ, part of the MALT, like PPs in the small gut. The B cell-dominant lymphocytes and myeloid cells are the commonest immune cells expressed in the tonsil. The surface epithelium follows the contours of the follicles and prolongs deeply into the tonsils to form crypts, highly increasing surface area (up to six times).

In the deepest part of the crypt, a lymphoepithelial symbiosis is created between the epithelium and the tonsil parenchyma. Here, there is an overexpression of antigen-presenting cells, such as M cells and dendritic cells, as well as memory B cells. Activated B cells differentiate into immunoblasts and produce antibodies by somatic hypermutation [18, 19].

MALT includes effector and inductive sites. PPs of the small intestine and tonsils are the most common inductive sites, where antigens prime naïve B cells through T cell-dependent and T cell-independent mechanisms [20]. T cell-independent pathway is stimulated by epithelial, dendritic, and stromal cells through the generation of some interleukins (like IL-6 and IL-10), transforming growth factor (TGF-β), B cell activating factor (BAFF or BLyS), and a proliferative inducing ligand (APRIL). In particular, BAFF and APRIL may bind the TNF receptor homolog transmembrane activator (TACI), stimulating the promotion of B cell differentiation and proliferation. Activated B cells translocate to regional lymph nodes via efferent lymphatics and systemic circulation, reaching the mucosal inductive sites, where they become effector cells [21]. Effector sites are present in all mucosa. In the T cell-dependent pathway, the B cell class switching happens after the antigen-specific T-cell activation. In PPs, IgA-secreting plasma cells generated from IgA+ plasmablasts start to produce dimeric IgA, formed by two IgA molecules linked by a joining chain [22]. The transcytosis of IgA dimers is mediated by binding to an epithelial glycoprotein, the polymeric Ig receptor (pIgR), expressed on the basolateral surface of the gut epithelium; IgA dimers cross the intestinal epithelial barrier and reach the apical surface, which, following the proteolytic cleavage of pIgR, are released into the lumen as secretory IgA. Innate immunity mechanisms operate in recognition of pathogens, particularly the stimulation of Toll-like receptors (TLRs), which promote the secretion of BAFF and enhance the B cell expression of histocompatibility complex class II molecules on B cells, promoting IgA production. Constant stimulation of TLRs may favor increased synthesis of Gd-IgA1 in prone subjects [23].

While in secretions polymeric IgA variants predominate, with equal representation of IgA1 and IgA2 subclasses, most IgA in circulation are produced in the bone marrow in monomeric IgA1 form. Polymeric IgA is prevalent in renal deposits of IgAN, and these IgA variants are increased in serum in 35–50% of patients with IgAN. Although these discoveries indicate a mucosal origin of the pathogenetic IgA in this disease, the predominance in deposits of IgA1 subclass, typical of bone marrow origin, remains unclear.

One intriguing hypothesis is that some mucosal IgA-secreting plasma cells could get lost during the migration from their mucosal induction sites to the bone marrow, possibly due to inappropriate expression of their surface homing receptors or, alternatively, faulty expression of mucosal homing counter-receptors on vascular endothelium, but its mechanism remains uncertain [24–26].

## The tonsil-kidney axis in IgAN

### Pathophysiology

The development of macroscopic hematuria in coincidence or shortly following an upper respiratory infection episode—the synpharyngitic hematuria—is a process still unclear at the glomerular level. The exact physio-pathological events leading to abrupt passage of a massive amount of red blood cells through the delicate glomerular structures without clear evidence of patent breaks are unknown [27]. However, there are no doubts to the hypothesis of the involvement of tonsillar NALT stimulation by microbes leading to a sharp increased production of IgA1 [28].

The first investigation on the relationship between IgAN and tonsils was performed by Tomino in 1983 [29]. This study reported a specific binding of IgA eluted from kidney samples of IgAN patients to tonsillar cells, suggesting a tonsillar origin of deposited IgA. Under microbe trigger, B cells can be stimulated, differentiated, and proliferated in germinal centers of tonsils, leading to polymeric IgA synthesis. Patients with IgAN have high levels of IgA-producing plasma cells in the tonsils, higher than controls, and even higher than subjects with recurrent tonsillitis [30]. These plasma cells originate from memory cells, with sustained production of IgA antibodies over the long term, and may migrate to the bone marrow and synthesize and release Gd-IgA in circulation.

Several studies have been devoted to investigating a role of specific microbes stimulating the NALT in patients with IgAN. Most pathogens can induce, in some exposed animals, IgA mesangial deposits which do not persist for long and do not cause hematuria and/or proteinuria [23, 31]. However, stable IgA deposits and urinary abnormalities were caused in mice, with a defective mucosal tolerance, by intranasal administration followed by systemic challenge of the respiratory *Sendai virus* [32, 33].

The *Hemophilus parainfluenzae*, commonly present in the oral cavity, is stimulated in cultured mononuclear cells from tonsils the synthesis of IgA [34]. A microbiome study found a similarity in tonsillar microbes between IgAN patients and subjects with frequent tonsillitis [35] particularly for *Prevotella*, *Fusobacterium*, *Sphingomonas*, and *Treponema* spp. These microbes were highly represented and supposed to have a role in the pathogenesis of IgAN. A recent tonsillar microbiota analysis showed differences between patients with IgAN and both healthy (HC) and disease controls. The amounts of *Rahnella*, *Ruminococcus_g2*, and *Clostridium_g21* were substantially higher in IgAN patients than in HC [36]. However, other microbiome studies detected comparable bacterial flora in tonsillar crypts of patients and controls, indicating that the host reaction to these bacteria might be central in the development of IgAN more than a specific microbe challenge, possibly regulated by a genetic predisposition.

Recent data have showed a peculiar activation of TLR9, which identifies unmethylated DNA sequences in bacterial and viral DNA CpG-ODN, in the tonsils of patients with IgAN. TLR9 expression was linked with the disease activity and clinical course after tonsil removal surgery [37]. Moreover, the TLR9 genotype was correlated with histologic severity of IgAN. In peripheral mononuclear cells from tonsillectomized IgAN, due to tonsil focal repeated infections, an increased expression of TLR9mRNA was also observed [38]. The expression of APRIL is increased from TLR9 ligand CpG-ODN, and it is associated with the production of nephritogenic IgA. NALT-activated B cells can pass from inductive mucosal sites to systemic effector sites, comprising bone marrow, through adhesion molecules and chemokine/chemokine receptors. Human B cells infected with Epstein-Barr virus (EBV) secrete Gd-IgA1. It has recently been hypothesized that EBV-infected IgA+ cells may be the source of Gd-IgA1 which have homing receptors for targeting the upper respiratory tract. Moreover, the temporal sequence of racial-specific differences in Epstein-Barr virus infection may be supposed to explain the racial disparity in the prevalence of IgAN [39].

### Effects of tonsillectomy

Several data support an association between NALT and IgAN. From a genetic background, it is of interest that the variant rs2412971, intronic in HOMARD2, was found to be linked with tonsillectomy in the general population and to increased risk of IgAN [40]. Tonsillectomy decreased serum and salivary IgA, particularly in children [41]. However, other results have been conflicting [42, 43]. Coppo’s group investigated a subgroup of patients with IgAN in the European VALIGA study who underwent tonsillectomy due to recurrent tonsillitis [44, 45]. The study reported that tonsillectomy did not affect the stimulation of innate immunity through TLRs and ubiquitin-proteasome pathways and the pro-oxidative milieu, even though the levels of Gd-IgA1 were lower in tonsillectomized patients with IgAN. The remaining stimulation of innate immunity in patients without tonsils was suggestive of an extra-tonsillar MALT activation in IgAN [38].

In several cases, tonsillar mucosal immunity has a role in the first manifestation of the disease with an evident link between upper respiratory tract infection and macroscopic hematuria. It is harder to prove its responsibility in the evolution of IgAN; indeed, the reduction of kidney function is not so closely affected by tonsillectomy as it should be estimated following this thesis [43].

In Japanese subjects, the advantage of tonsil removal surgery, primarily asserted as an independent factor for progression of IgAN to ESKD [42], has been recently confirmed typically together with steroids, which are likely to have the major curative aspect [46].

However, recent KDIGO-reviewed guidelines (to be published in 2021) consider different benefits of tonsillectomy in different ethnicities. Hence, these line guides recommend that tonsillectomy should not be performed as a treatment for IgAN in Caucasian patients, with the exception of cases with recurrent tonsillitis. In Japanese patients, several cohort studies including a large retrospective study with propensity matching analysis reported improved kidney survival following tonsillectomy [47]. A single RCT comparing tonsillectomy and corticosteroids pulses versus corticosteroid pulses alone failed to show beneficial effect in the attenuation of hematuria or incidence of clinical remission at 1 year.

This led to the conclusion that in many patients with IgAN, particularly Caucasians, tonsillectomy does not show to influence the remission or a better outcome of the disease. A marginal beneficial effect in Japanese ethnicity may be considered.

## The gut-kidney axis in IgAN: two faces of the same coin

### The gut-kidney axis in IgAN: between genes and environment

The function of the microbiota and mucosal immunity in the development of IgAN rests central [48], although searching for specific mucosal microorganisms promoting IgA synthesis has been inexhaustible [23]. Advancement in the field of knowledge on the role played by gut microbiota exposure in patients susceptible to developing IgAN was proposed by genome-wide association studies (GWAS) [49]. GWAS are methods that provide a complete analysis of the total genome in order to identify genetic regions (loci) associated at risk of developing the disease [50]. The most important milestone in IgAN GWAS studies belongs to the Gharavi group [49]. In this study, the authors identified a relationship between the genetic probability to develop IgAN and climatic, pathogenic load, and dietary elements; nevertheless, the strongest positive correlation was showed between the local pathogen diversity (including viruses, bacteria, protozoa, and helminths) and the score of genetic risk for IgAN. The increased prevalence of IgAN in some regions might be the effect of a defensive adaptation from gut worm mucosal invasion. On the other hand, IgAN susceptibility loci were related with the predisposition to inflammatory bowel diseases (IBD), with the proteins implicated in the protection of the gut integrity and in the control of the mucosal immune response of the gut environment. In particular, these genetic risk alleles significantly impact the age at onset of the disease. The conclusion was that the striking association between genetics and environmental elements could induce the functional changes into the gut mucosal immune system favoring the onset of the disease.

### The gut-kidney axis in IgAN: a “microbiotic” view on IgAN

The intestinal microbiota and its metabolites (the metabolome) have been indicated to have a key role on immune balance [51]. It has been assessed that the human gastrointestinal system comprises up to 10^14^ bacteria with a biomass of 2 kg. These gut microbiota are in contact with MALT and are involved in maintaining the intestinal permeability and the immune system [52]. The gut microbiota is known to be implicated in the host innate and adaptive immune system; mutually, the composition of the gut flora depends on intestinal immune system that defends against pathogens through the production of IgA. In particular, numerous studies indicate that the microbial infections stimulate the differentiation of B-cells into IgA-secreting plasma cells, through T cell–independent or T cell–dependent pathways [53, 54]. However, the precise correlation between Gd-IgA and gut microbiota in patients with IgAN is uncertain.

It has been theorized that the release of intestinal microbiome products, such as lipopolysaccharide (LPS) and lipoteichoic acid, may activate GALT through TLR pathways. Indeed, LPS is a ligand for TLR4; lipoteichoic acid, for TLR2 [55]. Microbiota signal via TLRs may modulate gut microbe challenge, mucosal injury, and repair [56]. Modifications in intestinal barrier, with increased intestinal permeability documented in patients with IgAN [57, 58], may facilitate LPS absorption and blood circulation. Bacterial LPS stimulates the activation of TLR4 in cultured peripheral B cells causing a methylation of Cosmc, leading to the defective galactosylation of IgA1 [59]. The TLR4 and the membrane CD14 (the receptor for complexes of LPS and LPS binding protein) are the main elements implicated in cellular LPS signaling, and CD14/-159 polymorphism was found to be correlated with progressive incidents of IgAN [60]. Coppo’s group reported that children and adults with IgAN and IgA vasculitis [61, 62] had greater levels of TRL4 mRNA in peripheral blood lymphomononuclear cells, which was associated with the activation of mucosal immunity and clinical signs. These observations suggest that raised gut permeability to intestinal microbes’ triggers, through activation of TLR4, may modulate the immune response in IgAN.

The interaction between MALT and gut flora, in the promotion of experimental IgAN, was showed in a transgenic mouse model overexpressing BAFF [20]. These mice with B cell hyperplasia show an increase in all Ig classes including IgA [63]. The overexpression of BAFF in this animal model is correlated with high expression of polymeric hypogalactosylated IgA and IgA renal deposits. The development of the disease was dependent on the microbiota, leading to the theory that an overexpression of BAFF signaling modifies the normal equilibrium with the commensal flora and alters the systemic immune response.

Gesualdo et al. conducted the first human study that showed a correlation between gut dysbiosis and IgAN [64]. In this cross-sectional study, they investigated the fecal microbiota, and the fecal and urinary metabolome of non-progressor (NP) and progressor (P) IgAN patients. Patients with progressive disease showed the lowest microbial diversity compared to NP and HC. At genera/species levels, *Ruminococcaceae*, *Lachnospiraceae*, *Eubacteriaceae*, and *Streptococcaeae* raised in the stool samples of NP and P patients result in an increase of *Firmicutes*, while HC showed higher abundance of *Clostridium*, *Enterococcus*, and *Lactobacillus* genera. Compared with HC, *Bifidobacterium* species decreased in the fecal samples of NP and P, while *Sutterellaceae* and *Enterobacteriaceae* species showed an opposite trend. Along the same lines, Sun et al. [65] have suggested the prospective role of intestinal microbiota as a specific biomarker and an actor in the diagnosis and pathogenesis of IgAN. Indeed, the authors demonstrated a substantial difference in the gut microbiota not only between IgAN patients and HC but also between IgAN patients and patients with membranous nephropathy (MN). The abundance of *Escherichia-Shigella*, consistent with the previous study, and *Defluviitaleaceae_incertae_sedis* were higher in IgAN than those in HC, while lower levels were found for *Roseburia*, *Lachnospiraceae_unclassified*, *Clostridium_sensu_stricto_1*, *Haemophilus*, and *Fusobacterium*. Moreover, in IgAN patients, the level of *Megasphaera* and *Bilophila* was higher, whereas that of *Megamonas*, *Veillonella*, *Klebsiella*, and *Streptococcus* was lower compared with those with MN. In addition, the authors correlated the microbiota with the clinical factors. The analysis demonstrated that in IgAN patients, *Prevotella* was positively correlated with the level of serum albumin, while *Klebsiella*, *Citrobacter*, and *Fusobacterium* were negatively correlated. Moreover, a positive correlation was showed between *Bilophila* and the presence of kidney crescents in the Oxford classification of IgAN.

Another step toward understanding the tight link between microbiota and IgAN is represented by one of the latest papers of Gesualdo’s group [54]. They showed that the modification of mucosal immunity, due to a change of the gut flora, has a key role in the development of the disease. Indeed, they found that IgAN patients had increased serum levels of BAFF and that it was positively correlated with amounts of five specific microbiota metabolites (4-(1,1,3,3-tetramethylbutyl) phenol, p-tert-butyl-phenol, methyl neopentyl phthalic acid, hexadecyl ester benzoic acid, and furanone A). Phenol exerts a toxic effect against the gut lumen; indeed, it is able to reduce barrier function, and to increase gut permeability [66], leading to mucosal hyper-responsivity. They also showed that IgAN patients have a higher level of circulating gut-homing (CCR9+ β7 integrin+) regulatory B cells, memory B cells, and IgA+ memory B cells compared with HC that predisposes to an atypical synthesis of Gd-IgA.

The close connection between microbiota and IgAN opens up a new field of therapeutic strategies in the gut microbiota manipulation, such as the use of dietary interventions, or antibiotics, prebiotics, and probiotics, or through fecal microbiota transplantation (FMT).

## New prospective therapy targeting the gut-mucosal immune system

### Food antigens

Increased gut permeability has been described in subjects with IgAN [58] and high levels of IgA anti-alimentary antigens were found in circulation [67]. Case reports of association between celiac disease (CD) and IgAN suggested a potential role for gluten [68]. In CD, an autoimmune inflammatory disease, gluten ingestion elicits zonulin overexpression, followed by tight junction disassembly and increased intestinal permeability. Gliadin can cross the gut barrier due to leaky gut and/or retro transcytosis of secretory IgA via transferrin receptor 1 (TfR1/CD71) and transglutaminase 2 (TG2). It elicits the B cell response and, consequently, the synthesis of antigliadin and TG2 antibodies. IgA-CIC containing gliadin can be deposited in the mesangium via TfR1 and TG2 [69].

This association was recently confirmed in 223 IgAN patients that showed a raised risk of CD (4% vs 0.5–1%) in the absence of celiac-type HLA DQ2-DQ8 [70]. On the other hand, in 27,160 patients with CD, an increased probability of IgAN (0.026% vs 0.008%, HR 3.03; CI 1.22–7.56) was demonstrated [71].

In a pioneering study, Coppo et al. examined BALB/c mice fed with gluten-free diet from birth for the effects of gluten and its lectin fraction gliadin [72]. Greater IgA deposits were found in mice fed with a gluten-rich diet compared to mice maintained on gluten-free diet. Serum and renal deposition of anti-gliadin antibodies was detected in mice on gluten-rich diet, suggesting a gluten-induced experimental IgAN. Recently, in a transgenic animal model in which the expression of both human IgA1 and human CD89 (α1KI-CD89TG) was induced, a gluten-free diet for three generations, to produce gluten sensitivity, lowered renal human IgA1 deposition, glomerular inflammation, and then TfR1 and TG2 expression, as well as hematuria. Lack of IgA1-sCD89 CIC in serum and renal eluates was found in mice on a gluten-free diet, while a gluten diet exacerbated gut IgA1 secretion, inflammation, and damage, and was associated with increased serum IgA1 anti-gliadin antibodies, and with the development of proteinuria. Likewise, early administration of gluten-free diet to transgenic mice prevented renal IgA1 deposition and hematuria. A rise in IgA1–sCD89 CIC, IgA1 renal deposition, and in IgA1 anti-gliadin antibodies was showed after a new challenge with gluten diet for 30 days, associated to gut damage (inflammation and villous atrophy). Therefore, the interaction between the gliadin and CD89 may be the cause of an exacerbation of IgAN through the generation of IgA1–sCD89 CIC and a stimulation of mucosal immune response [73–75].

The effect of a gluten-free diet reported in the elegant experimental model of IgAN in double transgenic mice is similar to what Coppo et al. reported several years ago in pilot studies conducted on patients with IgAN [76]. Gluten-free for two periods of 1 month and 6 months in patients with IgAN negative for a subclinical celiac disease was related with a significant reduction in IgA containing immune complexes, with rebounds after intervals of 1 and 3 months of gluten-free diet. A gluten-free diet can lower the serum concentration of IgA directed against alimentary components (β-lactoglobulin, casein, ovalbumin), thus interrupting a gluten-dependent high gut permeability. In a pilot study on IgAN patients with no sign of CD, a reduction of proteinuria and hematuria was noticed, and no decline of kidney function was found over a period of 4 years [77, 78]. All patients had no intestinal symptoms before or after a gluten-free diet.

Recently, gluten sensitivity in patients affected by IgAN attracted new attention, after the discovery of a key role of gluten in inducing the production of high abundance of IgA anti-gliadin and mucosal reactivity [79, 80]. A rectal mucosal patch with gliadin induced in 30% of patients with IgAN higher production of nitric oxide and of myeloperoxidase as well as increasing IgA anti gliadin and several alimentary components.

A gluten-free diet is commonly adopted for CD, but interest has been recently devoted to a gluten-free diet in non-celiac wheat sensitivity (NCWS), a non-allergic and non-immune disorder with gut and extra-gut symptoms that resolve after a gluten-free diet [81, 82]. The prevalence of NCWS is 3–6% of the general population. The diagnosis of NCWS is made on the benefits from a gluten-free diet for 6 weeks and recurrence after 2 weeks on a gluten-containing diet. These subjects have no association with HLA DQ8- HLA DQ2 (celiac haplotype). It is of interest to note that a gluten-free diet is under study in some registered RCTs on diseases that do not present with NCWS, including type 1 diabetes and schizophrenia. A RCT is just completed and results are expected about microbiome composition changes on a 4-week gluten-free diet challenge. No RCT adopting a gluten-free diet in IgAN is registered.

### Probiotics

The use of probiotics has been found to have anti-inflammatory and anti-oxidative effects, and it is able to modify the intestinal microbiota; moreover, it has been efficient in preventing enteric infections and inflammatory and malignant diseases [83]. In nephrological context, the consumption of probiotics, prebiotics, or symbiotics could play a central role on kidney health [84]. In chronic kidney disease (CKD) probiotics may improve the barrier function leading to a reduction of uremic toxins, blood urea nitrogen, oxidative stress, and markers of inflammation [85].

The dietary programs with a high prebiotic load have been successfully used to modulate the microbiota and could be a new and low-risk restorative approach in the management of IgAN. On these bases, the consistency of this “proof of concept” was described by Soylu et al. The authors proved that the administration of *Saccharomyces boulardii*, a gram-positive yeast with probiotic properties used in diarrhea treatment, successfully decreased systemic IgA production, inducing protection against the disease in IgAN mice. The probable mechanisms underlying these results include the decrease of gut inflammation, via modulation of the T cell, inhibition of pathogens, hindering the adherence of the pathogens to the intestinal mucosa, inhibition of the microbial toxins, production of IgA, and trophic effects on gut wall [86].

### Antibiotics

Another approach in manipulating the gut microbiota is represented by the use of antibiotics. In this scenario, a new study conducted by Monteiro et al. [87] revealed that, in a humanized mouse model of IgAN, the use of broad-spectrum antibiotics (vancomycin/amoxicillin/neomycin/metronidazole) could abolish mesangial IgA1 deposits and proteinuria; moreover, it can also affect the concentration of hIgA1–mIgG immunocomplexes in the circulatory system. However, given the risk of collateral effects of antibiotic therapy, such as *Clostridium difficile* infections and antibiotic resistance, it should be used with caution [88].

To date, new potential therapeutic strategies have been emerging. Therefore, considering the role of the intestinal microbiota in the IgAN, scientists have created programmed inhibitor cells (PICs) that exert the antibacterial activity against selective and specified species or strains of bacteria. The PICs express surface-displayed nanobodies that mediate antigen-specific cell–cell adhesion to overcome the barrier to T6SS (type VI secretion system) activity in liquid medium. This kind of intervention has been suggested to not be associated with damage to complex microbial communities, with rare resistance [89]. However, although some researches have been conducted, further study about these topics is still needed for medical application.

### Fecal microbiota transplantation

Excepting the use of prebiotics and antibiotics, numerous new weapons for restoring the gut microbiota, such as FMT, have begun to be developed.

More specifically, this approach is indicated as the most successful treatment in patients with *Clostridium difficile* infection and it is a fecal transfer from a selected healthy subject to the recipient’s gastrointestinal tract experiencing microbial dysbiosis [90].

Currently, there are no strong data confirming the utilization of the FMT in IgAN; it is proven that, in patients suffering from CKD, FMT through a positive action on intestinal microbiota diversity limits the increase of uremic toxins released from the gut cresol pathway [91].

Promising clinical trials are aiming at disclosing whether this option may have a role in the treatment of IgAN. In fact, an interventional study (clinical trial NCT03633864) is currently being conducted and intends to define the security and efficiency of FMT in IgAN subjects resistant to the standard therapy.

### Enteric budesonide

IgAN represents the commonest glomerular disease, found on kidney biopsy, in patients with IBD, supporting a close association between the immune mechanisms subtending IBD and IgAN [92].

A recently published study, conducted on a Swedish population, compared 3963 cases of patients with IgAN and 20,000 matched controls [93]. During a median follow-up of 13 years, IBD developed in 4.95% patients with IgAN versus 1.65% matched. The diagnosis of IBD was more common before a confirmed IgAN diagnosis; also IgAN patients had an increased risk to develop IBD compared with controls. Moreover, both logistic regression and time-varying Cox regression demonstrated that IBD elevates the ESKD risk in patients with IgAN.

This correlation suggested the possible benefits of budesonide, a drug used mainly in IBD that targets the intestinal immunity and local inflammation in the gut mucosa and in PPs [94, 95]. To this end, the ileum targeting-release formulation of the glucocorticosteroid budesonide (TRF budesonide; Nefecon™) is an emerging class of immunosuppressants created to deliver the drug to the upper ileum (where PPs are mostly represented) and acts on the local immune hyperresponsiveness, reducing the systemic adverse effects (only 10% reaches systemic circulation).

In a preliminary study, conducted on sixteen IgA patients, 8 mg/day of budesonide was given for 6 months, followed by a 3-month follow-up period; enteric budesonide was able to reduce the urine albumin excretion as well as, albeit slightly, the serum creatine [96].

In a phase 2b trial (NEFIGAN a randomized, double-bind, placebo-controlled study), two Nefecon regimens (8 mg/day and 16 mg/day) were tested on 149 IgAN patients. At 9 months, TRF-budesonide was associated with significant reduction of urine protein creatinine ratio (uPCR): −27.3% in 48 patients who received 16 mg/day and −21.5% in the 51 patients who received 8 mg/day (−24.4% from baseline in the two Nefecon groups combined) versus an increase of 2.7% in the placebo arm [95, 97].

However, the phase 2 study enrolled a limited number of subjects, followed for a relatively short time and randomized to be almost exclusively Caucasian; thus, the generalizability of the findings to other ethnicities has to be confirmed. Meanwhile, a phase 3 multicenter, randomized, double-blind, placebo-controlled study (NEFIGARD trial: NCT 03643965) is currently underway, and it aims to evaluate the safety and efficacy of TRF-budesonide (at a dose of 16 mg/day) compared with placebo in patients with a histological diagnosis of primary IgAN on a background of optimized RAS inhibitor therapy.

### Hydroxychloroquine

Another interesting therapeutic weapon targeting the MALT is the hydroxychloroquine (HCQ) that can have a beneficial effect on IgAN patients.

Indeed, the HCQ, inhibiting the mucosal and intrarenal TLRs signaling, is able to reduce the cytokine and chemokine production as well as to suppress the presentation of autoantigens, thus exercising immunomodulatory and anti-inflammatory actions [98, 99].

The first phase 2, double-blind, randomized, placebo controlled clinical trial has newly been published [100]. The study performed by Liu et al. tested oral HCQ in IgAN patients receiving maximal supportive treatment, including RAAS inhibitor therapy and blood pressure control. A significant decrease in proteinuria (48.4%) was showed in the HCQ group compared to a 10% increase in the placebo group without differences in the percentage of change of eGFR after only 6 months of follow-up [99, 101].

### Emerging therapies

Currently, there are some ongoing trials that are testing some emerging drugs able to interfere with the T cell–independent pathway and endothelin receptor. More specifically, atacicept (a dual BAFF/APRIL inhibitor) reduces the serum levels of IgA in patients blocking the activation of TACI, while blisibimod is a BAFF antagonist; both are under evaluation in separate phase II studies in patients with IgAN (Clinical Trial nos. NCT02808429 and NCT02062684). Likewise, VIS649, a humanized anti-APRIL antibody, was also described to reduce the IgA serum levels in non-human primates [102].

Moreover, B cell depletion therapy (e.g., bortezomib, rituximab, and obinutuzumab) aims to control the autoimmune response and could be a possible choice for future therapy; indeed, some researchers already included it in alternative weapons (NCT02571842) [103, 104].

Finally, the use of sparsentan (a dual blocker of angiotensin II and endothelin 1 receptors) to decrease proteinuria and stabilize eGFR is ongoing in a phase III trial.

## Conclusions

Mucosal immunity has a key role in the pathogenesis of IgAN by multiple mechanisms, mostly active at the gut-kidney axis. Genetic, environment, and dietary elements are able to cooperate in causing functional changes of the gut mucosal immune system promoting the development and progression of the disease. Innovative therapeutic approaches may have a future impact on the treatment of this disease.

## References

[1] Gharavi AG, Yan Y, Scolari F, Schena FP, Frasca GM, Ghiggeri GM, Cooper K, Amoroso A, Viola BF, Battini G, Caridi G, Canova C, Farhi A, Subramanian V, Nelson-Williams C, Woodford S, Julian BA, Wyatt RJ, Lifton RP (2000). IgA nephropathy, the most common cause of glomerulonephritis, is linked to 6q22-23. Nat Genet.

[2] Berger J (1969). IgA glomerular deposits in renal disease. Transplant Proc.

[3] Miura M, Tomino Y, Endoh M, Suga T, Kaneshige H, Nomoto Y, Sakai H (1982). A case of IgA nephropathy associated with marked hematuria after upper respiratory tract infections. Tokai J Exp Clin Med.

[4] Seikrit C, Rauen T, Floege J (2019). Immunoglobulin A nephropathy. Internist (Berl).

[5] Monteiro RC (2018). Recent advances in the physiopathology of IgA nephropathy. Nephrol Ther.

[6] Placzek WJ, Yanagawa H, Makita Y, Renfrow MB, Julian BA, Rizk DV, Suzuki Y, Novak J, Suzuki H (2018). Serum galactose-deficient-IgA1 and IgG autoantibodies correlate in patients with IgA nephropathy. PLoS One.

[7] Suzuki H, Kiryluk K, Novak J, Moldoveanu Z, Herr AB, Renfrow MB, Wyatt RJ, Scolari F, Mestecky J, Gharavi AG, Julian BA (2011). The pathophysiology of IgA nephropathy. J Am Soc Nephrol.

[8] Suzuki H (2019). Biomarkers for IgA nephropathy on the basis of multi-hit pathogenesis. Clin Exp Nephrol.

[9] Yan Y, Xu LX, Zhang JJ, Zhang Y, Zhao MH (2006). Self-aggregated deglycosylated IgA1 with or without IgG were associated with the development of IgA nephropathy. Clin Exp Immunol.

[10] Coppo R (2015). The intestine-renal connection in IgA nephropathy. Nephrol Dial Transplant.

[11] Coppo R (2017). C4d deposits in IgA nephropathy: where does complement activation come from?. Pediatr Nephrol.

[12] Maillard N, Wyatt RJ, Julian BA, Kiryluk K, Gharavi A, Fremeaux-Bacchi V, Novak J (2015). Current understanding of the role of complement in IgA nephropathy. J Am Soc Nephrol.

[13] Bienenstock, KCAJ (1994) Characteristics and functions of mucosa-associated lymphoid tissue. Handbook of mucosal immunology 141-149

[14] Gormley PD, Powell-Richards AOR, Azuara-Blanco A, Donoso LA, Dua HS (1998). Lymphocyte subsets in conjunctival mucosa-associated-lymphoid-tissue after exposure to retinal-S-antigen. Int Ophthalmol.

[15] Kiyono H, Fukuyama S (2004). NALT- versus Peyer’s-patch-mediated mucosal immunity. Nat Rev Immunol.

[16] Randall TD, Mebius RE (2014). The development and function of mucosal lymphoid tissues: a balancing act with micro-organisms. Mucosal Immunol.

[17] Kimura S (2018). Molecular insights into the mechanisms of M-cell differentiation and transcytosis in the mucosa-associated lymphoid tissues. Anat Sci Int.

[18] Cesta MF (2006). Normal structure, function, and histology of mucosa-associated lymphoid tissue. Toxicol Pathol.

[19] Harabuchi Y, Takahara M (2019). Recent advances in the immunological understanding of association between tonsil and immunoglobulin A nephropathy as a tonsil-induced autoimmune/inflammatory syndrome. Immun Inflamm Dis.

[20] Fagarasan S, Kawamoto S, Kanagawa O, Suzuki K (2010). Adaptive immune regulation in the gut: T cell-dependent and T cell-independent IgA synthesis. Annu Rev Immunol.

[21] Xin G, Shi W, Xu LX, Su Y, Yan LJ, Li KS (2013). Serum BAFF is elevated in patients with IgA nephropathy and associated with clinical and histopathological features. J Nephrol.

[22] Gutzeit C, Magri G, Cerutti A (2014). Intestinal IgA production and its role in host-microbe interaction. Immunol Rev.

[23] Rollino C, Vischini G, Coppo R (2016). IgA nephropathy and infections. J Nephrol.

[24] Knoppova B (2016). The origin and activities of IgA1-containing immune complexes in IgA nephropathy. Front Immunol.

[25] Coppo R (2018). Treatment of IgA nephropathy: recent advances and prospects. Nephrol Ther.

[26] Yu HH, Chu KH, Yang YH, Lee JH, Wang LC, Lin YT, Chiang BL (2011). Genetics and immunopathogenesis of IgA nephropathy. Clin Rev Allergy Immunol.

[27] Yuste C, Gutierrez E, Sevillano AM, Rubio-Navarro A, Amaro-Villalobos JM, Ortiz A, Egido J, Praga M, Moreno JA (2015). Pathogenesis of glomerular haematuria. World J Nephrol.

[28] Feehally J, Beattie TJ, Brenchley PEC, Coupes BM, Mallick NP, Postlethwaite RJ (1986). Sequential study of the IgA system in relapsing IgA nephropathy. Kidney Int.

[29] Tomino Y, Sakai H, Endoh M, Suga T, Miura M, Kaneshige H, Nomoto Y (1983). Cross-reactivity of IgA antibodies between renal mesangial areas and nuclei of tonsillar cells in patients with IgA nephropathy. Clin Exp Immunol.

[30] Bene MC (1993). Tonsils in IgA nephropathy. Contrib Nephrol.

[31] Coppo R, Amore A, Peruzzi L, Vergano L, Camilla R (2010). Innate immunity and IgA nephropathy. J Nephrol.

[32] Amore A, Coppo R, Nedrud JG, Sigmund N, Lamm ME, Emancipator SN (2004). The role of nasal tolerance in a model of IgA nephropathy induced in mice by Sendai virus. Clin Immunol.

[33] Gesualdo L, Lamm ME, Emancipator SN (1990). Defective oral tolerance promotes nephritogenesis in experimental IgA nephropathy induced by oral immunization. J Immunol.

[34] Ogura Y, Suzuki S, Shirakawa T, Masuda M, Nakamura H, Iijima K, Yoshikawa N (2000). Haemophilus parainfluenzae antigen and antibody in children with IgA nephropathy and Henoch-Schonlein nephritis. Am J Kidney Dis.

[35] Watanabe H (2017). Comprehensive microbiome analysis of tonsillar crypts in IgA nephropathy. Nephrol Dial Transplant.

[36] Park JI, Kim TY, Oh B, Cho H, Kim JE, Yoo SH, Lee JP, Kim YS, Chun J, Kim BS, Lee H (2020). Comparative analysis of the tonsillar microbiota in IgA nephropathy and other glomerular diseases. Sci Rep.

[37] Muto M, Manfroi B, Suzuki H, Joh K, Nagai M, Wakai S, Righini C, Maiguma M, Izui S, Tomino Y, Huard B, Suzuki Y (2017). Toll-like receptor 9 stimulation induces aberrant expression of a proliferation-inducing ligand by tonsillar germinal center B cells in IgA nephropathy. J Am Soc Nephrol.

[38] Vergano L, Loiacono E, Albera R, Coppo R, Camilla R, Peruzzi L, Amore A, Donadio ME, Chiale F, Boido A, Mariano F, Mazzucco G, Ravera S, Cancarini G, Magistroni R, Beltrame G, Rollino C, Stratta P, Quaglia M, Bergia R, Cravero R, Cusinato S, Benozzi L, Savoldi S, Licata C (2015). Can tonsillectomy modify the innate and adaptive immunity pathways involved in IgA nephropathy?. J Nephrol.

[39] Zachova K, Kosztyu P, Zadrazil J, Matousovic K, Vondrak K, Hubacek P, Julian BA, Moldoveanu Z, Novak Z, Kostovcikova K, Raska M, Mestecky J (2020). Role of Epstein-Barr Virus in pathogenesis and racial distribution of IgA nephropathy. Front Immunol.

[40] Feenstra B, Bager P, Liu X, Hjalgrim H, Nohr EA, Hougaard DM, Geller F, Melbye M (2017). Genome-wide association study identifies variants in HORMAD2 associated with tonsillectomy. J Med Genet.

[41] D'Amelio R (1982). Serum and salivary IgA levels in normal subjects: comparison between tonsillectomized and non-tonsillectomized subjects. Int Arch Allergy Appl Immunol.

[42] Xie Y, Nishi S, Ueno M, Imai N, Sakatsume M, Narita I, Suzuki Y, Akazawa K, Shimada H, Arakawa M, Gejyo F (2003). The efficacy of tonsillectomy on long-term renal survival in patients with IgA nephropathy. Kidney Int.

[43] Zand L, Fervenza FC (2014). Does tonsillectomy have a role in the treatment of patients with immunoglobulin A nephropathy?. Nephrol Dial Transplant.

[44] Coppo R, Troyanov S, Bellur S, Cattran D, Cook HT, Feehally J, Roberts ISD, Morando L, Camilla R, Tesar V, Lunberg S, Gesualdo L, Emma F, Rollino C, Amore A, Praga M, Feriozzi S, Segoloni G, Pani A, Cancarini G, Durlik M, Moggia E, Mazzucco G, Giannakakis C, Honsova E, Sundelin BB, di Palma AM, Ferrario F, Gutierrez E, Asunis AM, Barratt J, Tardanico R, Perkowska-Ptasinska A, on behalf of the VALIGA study of the ERA-EDTA Immunonephrology Working Group (2014). Validation of the Oxford classification of IgA nephropathy in cohorts with different presentations and treatments. Kidney Int.

[45] Feehally J, Coppo R, Troyanov S, Bellur SS, Cattran D, Cook T, Roberts ISD, Verhave JC, Camilla R, Vergano L, Egido J, Wiecek A, Karkoszka H, Tesar V, Maixnerova D, Ots-Rosenberg M, Quaglia M, Rollino C, Magistroni R, Cusinato S, Cravero R, Peruzzi L, Lundberg S, Gesualdo L, Cancarini G, Feriozzi S, Ferrario F, on behalf of the VALIGA study of the ERA-EDTA Immunonephrology Working Group (2016). Tonsillectomy in a European cohort of 1,147 patients with IgA nephropathy. Nephron.

[46] Kawamura T, Yoshimura M, Miyazaki Y, Okamoto H, Kimura K, Hirano K, Matsushima M, Utsunomiya Y, Ogura M, Yokoo T, Okonogi H, Ishii T, Hamaguchi A, Ueda H, Furusu A, Horikoshi S, Suzuki Y, Shibata T, Yasuda T, Shirai S, Imasawa T, Kanozawa K, Wada A, Yamaji I, Miura N, Imai H, Kasai K, Soma J, Fujimoto S, Matsuo S, Tomino Y, The Special IgA Nephropathy Study Group (2014). A multicenter randomized controlled trial of tonsillectomy combined with steroid pulse therapy in patients with immunoglobulin A nephropathy. Nephrol Dial Transplant.

[47] Hirano K, Matsuzaki K, Yasuda T, Nishikawa M, Yasuda Y, Koike K, Maruyama S, Yokoo T, Matsuo S, Kawamura T, Suzuki Y (2019). Association between tonsillectomy and outcomes in patients with immunoglobulin A nephropathy. JAMA Netw Open.

[48] Coppo R (2018). The gut-kidney axis in IgA nephropathy: role of microbiota and diet on genetic predisposition. Pediatr Nephrol.

[49] Kiryluk K, Li Y, Scolari F, Sanna-Cherchi S, Choi M, Verbitsky M, Fasel D, Lata S, Prakash S, Shapiro S, Fischman C, Snyder HJ, Appel G, Izzi C, Viola BF, Dallera N, del Vecchio L, Barlassina C, Salvi E, Bertinetto FE, Amoroso A, Savoldi S, Rocchietti M, Amore A, Peruzzi L, Coppo R, Salvadori M, Ravani P, Magistroni R, Ghiggeri GM, Caridi G, Bodria M, Lugani F, Allegri L, Delsante M, Maiorana M, Magnano A, Frasca G, Boer E, Boscutti G, Ponticelli C, Mignani R, Marcantoni C, di Landro D, Santoro D, Pani A, Polci R, Feriozzi S, Chicca S, Galliani M, Gigante M, Gesualdo L, Zamboli P, Battaglia GG, Garozzo M, Maixnerová D, Tesar V, Eitner F, Rauen T, Floege J, Kovacs T, Nagy J, Mucha K, Pączek L, Zaniew M, Mizerska-Wasiak M, Roszkowska-Blaim M, Pawlaczyk K, Gale D, Barratt J, Thibaudin L, Berthoux F, Canaud G, Boland A, Metzger M, Panzer U, Suzuki H, Goto S, Narita I, Caliskan Y, Xie J, Hou P, Chen N, Zhang H, Wyatt RJ, Novak J, Julian BA, Feehally J, Stengel B, Cusi D, Lifton RP, Gharavi AG (2014). Discovery of new risk loci for IgA nephropathy implicates genes involved in immunity against intestinal pathogens. Nat Genet.

[50] Sallustio F et al (2019) A new vision of IgA nephropathy: the missing link. Int J Mol Sci 21(1) 10.3390/ijms21010189 PMC6982283 31888082

[51] Bunker JJ, Bendelac A (2018). IgA responses to microbiota. Immunity.

[52] Floege J, Feehally J (2016). The mucosa-kidney axis in IgA nephropathy. Nat Rev Nephrol.

[53] Shreiner AB, Kao JY, Young VB (2015). The gut microbiome in health and in disease. Curr Opin Gastroenterol.

[54] Sallustio F et al (2020) High levels of gut-homing immunoglobulin A-positive+B lymphocytes support the pathogenic role of intestinal mucosal hyperresponsiveness in immunoglobulin A nephropathy patients. Nephrol Dial Transplant 10.1093/ndt/gfaa344 PMC8396395 33661282

[55] Yiu JH, Dorweiler B, Woo CW (2017). Interaction between gut microbiota and toll-like receptor: from immunity to metabolism. J Mol Med (Berl).

[56] Mazmanian SK, Liu CH, Tzianabos AO, Kasper DL (2005). An immunomodulatory molecule of symbiotic bacteria directs maturation of the host immune system. Cell.

[57] Davin JC, Forget P, Mahieu PR (1988). Increased intestinal permeability to (51 Cr) EDTA is correlated with IgA immune complex-plasma levels in children with IgA-associated nephropathies. Acta Paediatr Scand.

[58] Rostoker G, Wirquin V, Terzidis H, Petit-Phar M, Chaumette MT, Delchier JC, Belghiti † D, Lang P, Dubert JM, Meignan M, Lagrue G, Weil B (1993). Mucosal immunity in primary glomerulonephritis. III. Study of intestinal permeability. Nephron.

[59] Qin W, Zhong X, Fan JM, Zhang YJ, Liu XR, Ma XY (2008). External suppression causes the low expression of the Cosmc gene in IgA nephropathy. Nephrol Dial Transplant.

[60] Yoon HJ, Shin JH, Yang SH, Chae DW, Kim H, Lee DS, Kim HL, Kim S, Lee JS, Kim YS (2003). Association of the CD14 gene -159C polymorphism with progression of IgA nephropathy. J Med Genet.

[61] Coppo R, Camilla R, Amore A, Peruzzi L, DaprÃ V, Loiacono E, Vatrano S, Rollino C, Sepe V, Rampino T, Dal Canton A (2010). Toll-like receptor 4 expression is increased in circulating mononuclear cells of patients with immunoglobulin A nephropathy. Clin Exp Immunol.

[62] Donadio ME, Loiacono E, Peruzzi L, Amore A, Camilla R, Chiale F, Vergano L, Boido A, Conrieri M, Bianciotto M, Bosetti FM, Coppo R (2014). Toll-like receptors, immunoproteasome and regulatory T cells in children with Henoch-Schonlein purpura and primary IgA nephropathy. Pediatr Nephrol.

[63] McCarthy DD, Kujawa J, Wilson C, Papandile A, Poreci U, Porfilio EA, Ward L, Lawson MAE, Macpherson AJ, McCoy KD, Pei Y, Novak L, Lee JY, Julian BA, Novak J, Ranger A, Gommerman JL, Browning JL (2011). Mice overexpressing BAFF develop a commensal flora-dependent. IgA-associated nephropathy J Clin Invest.

[64] De Angelis M (2014). Microbiota and metabolome associated with immunoglobulin A nephropathy (IgAN). PLoS One.

[65] Dong R, Bai M, Zhao J, Wang D, Ning X, Sun S (2020). A comparative study of the gut microbiota associated with immunoglobulin a nephropathy and membranous nephropathy. Front Cell Infect Microbiol.

[66] Nyangale EP, Mottram DS, Gibson GR (2012). Gut microbial activity, implications for health and disease: the potential role of metabolite analysis. J Proteome Res.

[67] Coppo R, Amore A, Roccatello D, Gianoglio B, Molino A, Piccoli G, Clarkson AR, Woodroffe AJ, Sakai H, Tomino Y (1991). IgA antibodies to dietary antigens and lectin-binding IgA in sera from Italian, Australian, and Japanese IgA nephropathy patients. Am J Kidney Dis.

[68] Helin H, Mustonen J, Reunala T, Pasternack A (1983). IgA nephropathy associated with celiac disease and dermatitis herpetiformis. Arch Pathol Lab Med.

[69] Abbad L, Monteiro RC, Berthelot L (2020). Food antigens and transglutaminase 2 in IgA nephropathy: molecular links between gut and kidney. Mol Immunol.

[70] Collin P, Syrjanen J, Partanen J, Pasternack A, Kaukinen K, Mustonen J (2002). Celiac disease and HLA DQ in patients with IgA nephropathy. Am J Gastroenterol.

[71] Welander A, Sundelin B, Fored M, Ludvigsson JF (2013). Increased risk of IgA nephropathy among individuals with celiac disease. J Clin Gastroenterol.

[72] Coppo R (1989). Gluten-induced experimental IgA glomerulopathy. Lab Investig.

[73] Papista C, Lechner S, Ben Mkaddem S, LeStang MB, Abbad L, Bex-Coudrat J, Pillebout E, Chemouny JM, Jablonski M, Flamant M, Daugas E, Vrtovsnik F, Yiangou M, Berthelot L, Monteiro RC (2015). Gluten exacerbates IgA nephropathy in humanized mice through gliadin-CD89 interaction. Kidney Int.

[74] Berthelot L, Papista C, Maciel TT, Biarnes-Pelicot M, Tissandie E, Wang PHM, Tamouza H, Jamin A, Bex-Coudrat J, Gestin A, Boumediene A, Arcos-Fajardo M, England P, Pillebout E, Walker F, Daugas E, Vrtosvnik F, Flamant M, Benhamou M, Cogné M, Moura IC, Monteiro RC (2012). Transglutaminase is essential for IgA nephropathy development acting through IgA receptors. J Exp Med.

[75] Cambier A, Gleeson PJ, Flament H, le Stang MB, Monteiro RC (2021). New therapeutic perspectives for IgA nephropathy in children. Pediatr Nephrol.

[76] Coppo R, Basolo B, Rollino C, Roccatello D, Martina G, Amore A, Bongiorno G, Piccoli G (1986). Mediterranean diet and primary IgA nephropathy. Clin Nephrol.

[77] Coppo R, Roccatello D, Amore A, Quattrocchio G, Molino A, Gianoglio B, Amoroso A, Bajardi P, Piccoli G (1990). Effects of a gluten-free diet in primary IgA nephropathy. Clin Nephrol.

[78] Coppo R, Amore A, Roccatello D (1992). Dietary antigens and primary immunoglobulin A nephropathy. J Am Soc Nephrol.

[79] Smerud HK, Fellstrom B, Hallgren R, Osagie S, Venge P, Kristjansson G (2009). Gluten sensitivity in patients with IgA nephropathy. Nephrol Dial Transplant.

[80] Kloster Smerud H (2010). Gastrointestinal sensitivity to soy and milk proteins in patients with IgA nephropathy. Clin Nephrol.

[81] Serena G, D'Avino P, Fasano A (2020). Celiac disease and non-celiac wheat sensitivity: state of art of non-dietary therapies. Front Nutr.

[82] Galipeau HJ, Verdu EF (2014). Gut microbes and adverse food reactions: focus on gluten related disorders. Gut Microbes.

[83] Vitetta L, Vitetta G, Hall S (2018). Immunological tolerance and function: associations between intestinal bacteria, probiotics, prebiotics, and phages. Front Immunol.

[84] Cosola C et al (2018) Nutrients, nutraceuticals, and xenobiotics affecting renal health. Nutrients 10(7) 10.3390/nu10070808 PMC6073437 29937486

[85] Caggiano G, Cosola C, di Leo V, Gesualdo M, Gesualdo L (2020). Microbiome modulation to correct uremic toxins and to preserve kidney functions. Curr Opin Nephrol Hypertens.

[86] Maldonado Galdeano C, Cazorla SI, Lemme Dumit JM, Vélez E, Perdigón G (2019). Beneficial effects of probiotic consumption on the immune system. Ann Nutr Metab.

[87] Chemouny JM, Gleeson PJ, Abbad L, Lauriero G, Boedec E, le Roux K, Monot C, Bredel M, Bex-Coudrat J, Sannier A, Daugas E, Vrtovsnik F, Gesualdo L, Leclerc M, Berthelot L, Ben Mkaddem S, Lepage P, Monteiro RC (2019). Modulation of the microbiota by oral antibiotics treats immunoglobulin A nephropathy in humanized mice. Nephrol Dial Transplant.

[88] He JW, Zhou XJ, Lv JC, Zhang H (2020). Perspectives on how mucosal immune responses, infections and gut microbiome shape IgA nephropathy and future therapies. Theranostics.

[89] Ting SY, Martínez-García E, Huang S, Bertolli SK, Kelly KA, Cutler KJ, Su ED, Zhi H, Tang Q, Radey MC, Raffatellu M, Peterson SB, de Lorenzo V, Mougous JD (2020). Targeted depletion of bacteria from mixed populations by programmable adhesion with antagonistic competitor cells. Cell Host Microbe.

[90] Bibbo S et al (2020) Fecal microbiota transplantation: screening and selection to choose the optimal donor. J Clin Med 9(6) 10.3390/jcm9061757 PMC7356099 32517023

[91] Barba C et al (2020) Effects of fecal microbiota transplantation on composition in mice with CKD. Toxins (Basel) 12(12) 10.3390/toxins12120741 PMC7761367 33255454

[92] Ambruzs JM, Walker PD, Larsen CP (2014). The histopathologic spectrum of kidney biopsies in patients with inflammatory bowel disease. Clin J Am Soc Nephrol.

[93] Rehnberg J, Symreng A, Ludvigsson JF, Emilsson L (2021). Inflammatory bowel disease is more common in patients with IgA nephropathy and predicts progression of ESKD: a Swedish population-based cohort study. J Am Soc Nephrol.

[94] Robinson M (1997). Optimizing therapy for inflammatory bowel disease. Am J Gastroenterol.

[95] Coppo R, Mariat C (2020). Systemic corticosteroids and mucosal-associated lymphoid tissue-targeted therapy in immunoglobulin A nephropathy: insight from the NEFIGAN study. Nephrol Dial Transplant.

[96] Smerud HK, Barany P, Lindstrom K, Fernstrom A, Sandell A, Pahlsson P, Fellstrom B (2011). New treatment for IgA nephropathy: enteric budesonide targeted to the ileocecal region ameliorates proteinuria. Nephrol Dial Transplant.

[97] Fellstrom BC (2017). Targeted-release budesonide versus placebo in patients with IgA nephropathy (NEFIGAN): a double-blind, randomised, placebo-controlled phase 2b trial. Lancet.

[98] Willis R, Seif AM, McGwin G, Martinez-Martinez LA, González EB, Dang N, Papalardo E, Liu J, Vilá LM, Reveille JD, Alarcón GS, Pierangeli SS (2012). Effect of hydroxychloroquine treatment on pro-inflammatory cytokines and disease activity in SLE patients: data from LUMINA (LXXV), a multiethnic US cohort. Lupus.

[99] Gutierrez E (2020). A personalized update on IgA nephropathy: a new vision and new future challenges. Nephron.

[100] Liu LJ, Yang YZ, Shi SF, Bao YF, Yang C, Zhu SN, Sui GL, Chen YQ, Lv JC, Zhang H (2019). Effects of hydroxychloroquine on proteinuria in IgA nephropathy: a randomized controlled trial. Am J Kidney Dis.

[101] Floege J (2019). Antimalarials in IgA nephropathy: did our supportive therapy armamentarium just increase?. Am J Kidney Dis.

[102] Myette JR, Kano T, Suzuki H, Sloan SE, Szretter KJ, Ramakrishnan B, Adari H, Deotale KD, Engler F, Shriver Z, Wollacott AM, Suzuki Y, Pereira BJG (2019). A proliferation inducing ligand (APRIL) targeted antibody is a safe and effective treatment of murine IgA nephropathy. Kidney Int.

[103] Hartono C, Chung M, Perlman AS, Chevalier JM, Serur D, Seshan SV, Muthukumar T (2018). Bortezomib for reduction of proteinuria in IgA nephropathy. Kidney Int Rep.

[104] Lafayette RA, Canetta PA, Rovin BH, Appel GB, Novak J, Nath KA, Sethi S, Tumlin JA, Mehta K, Hogan M, Erickson S, Julian BA, Leung N, Enders FT, Brown R, Knoppova B, Hall S, Fervenza FC (2017). A randomized, controlled trial of rituximab in IgA nephropathy with proteinuria and renal dysfunction. J Am Soc Nephrol.

